# Community perceptions of intimate partner violence - a qualitative study from urban Tanzania

**DOI:** 10.1186/1472-6874-11-13

**Published:** 2011-04-18

**Authors:** Rose M Laisser, Lennarth Nyström, Helen I Lugina, Maria Emmelin

**Affiliations:** 1 Muhimbili University of Health and Allied Sciences (MUHAS), Midwifery School, PO Box 65006, Dar es Salaam, Tanzania; 2 Department of Public Health and Clinical Medicine, Epidemiology and Global Health, and Umeå Centre of Gender Studies, Umeå University, 901-85 Umeå, Sweden; 3 Bugando University College of Health Sciences, Archbishop Antony Mayalla School of Nursing, Mwanza Tanzania; 4 Department of Clinical Sciences, Social Medicine and Global Health, Lund University, PO Box 117, 22100 Lund, Sweden

## Abstract

**Background:**

Intimate partner violence against women is a prevailing public health problem in Tanzania, where four of ten women have a lifetime exposure to physical or sexual violence by their male partners. To be able to suggest relevant and feasible community and health care based interventions, we explored community members' understanding and their responses to intimate partner violence.

**Methods:**

A qualitative study using focus group discussions with 75 men and women was conducted in a community setting of urban Tanzania. We analysed data using a grounded theory approach and relate our findings to the ecological framework of intimate partner violence.

**Results:**

The analysis resulted in one core category, "Moving from frustration to questioning traditional gender norms", that denoted a community in transition where the effects of intimate partner violence had started to fuel a wish for change. At the societal level, the category "Justified as part of male prestige" illustrates how masculinity prevails to justify violence. At the community level, the category "Viewed as discreditable and unfair" indicates community recognition of intimate partner violence as a human rights concern. At the relationship level, the category "Results in emotional entrapment" shows the shame and self-blame that is often the result of a violent relationship. At the individual level, the risk factors for intimate partner violence were primarily associated with male characteristics; the category "Fed up with passivity" emerged as an indication that community members also acknowledge their own responsibility for change in actions.

**Conclusions:**

Prevailing gender norms in Tanzania accept women's subordination and justify male violence towards women. At the individual level, an increasing openness makes it possible for women to report, ask for help, and become proactive in suggesting preventive measures. At the community level, there is an increased willingness to intervene but further consciousness-raising of the human rights perspective of violence, as well as actively engaging men. At the macro level, preventive efforts must be prioritized through re-enforcement of legal rights, and provision of adequate medical and social welfare services for both survivors and perpetrators.

## Background

Intimate partner violence (IPV) is one form of gender-based violence that concerns people in intimate relationships. Those involved may be current or former spouses, boyfriends, or girlfriends in heterosexual or homosexual relationships [1]. The perpetrators can be either men or women [2]. However, this paper concentrates on IPV by men against women because of its commonness and the serious negative effects on a woman's health.

IPV by men against women is a worldwide public health and human rights concern. According to the WHO multi-country study, performed in 10 countries using a standardized methodology, the prevalence of different types of IPV vary between 15% and 71% among women aged 15-49 years [3,4]. Studies from developing countries that were not involved in the WHO study, such as Haiti, Nigeria and Uganda, have estimates with a similar variation (11-52%) [5-7]. In urban Dar es Salaam, Tanzania, estimates of lifetime prevalences are 33% for physical and 23% for sexual violence against women [4]. In the WHO study, physical violence included actions such as being beaten, hit, kicked, choked, burned or threatened with a weapon by a current or former partner/husband. Sexual violence was defined as being physically forced or threatened to have sex or to do something sexually degrading.

IPV against women is associated with an increased risk for health problems [8]. This affects the economy since women's productivity is reduced [9]. IPV is a direct cause of physical and psychological injuries [4] and five times more often perpetrated by men against women than vice versa [10,11]. More than 25% of the women in the WHO multi-county study suffered severe injuries such as fractures and broken teeth [4]. Psychological problems such as depression, anxiety and post traumatic stress disorders are other common health effects, as are gynaecological and other reproductive health problems, adverse pregnancy outcomes, chronic pain, and changes in endocrine and immune functions [12-14]. IPV is known to be associated with feelings of shame, guilt and poor self esteem [15]. Abused women seek medical care three times more often than those who are not abused [8], indicating that they have more ill health compared to unaffected women. Abused women have reduced coping capacity that may lead to alcohol or drug abuse, suicidality, homicides, maternal mortality and HIV infection [4].

Several studies have indicated a high risk of IPV against women in male-dominant, patriarchal societies where gender attitudes and perceptions support marked inequality between men and women and where rigid gender roles may lead to justification and acceptance of IPV [5,16-20]. In countries where women are economically dependent, the challenge of reducing violence is reported to be even greater. Studies from Ethiopia, Zambia and Kenya have found poverty, low level of education and unemployment among women to increase the risk of IPV [21-23].

Socio-cultural norms and judiciary systems [24] often make it difficult for women to leave violent partners. In many settings religious norms cause further constraints since they often include strong beliefs that marriage should be maintained at any cost, even if divorce is accepted by civil law [19]. Negative attitudes from the police, financial dependency, family ties and consideration for the children put additional pressure on women to stay in violent relationships [17,25-28].

Traditional gender constructions restrict women's influence and activities to the household level where domestic labour, childbearing, and child rearing dominate. In contrast, men are given the higher valued task of being the bread winner. Societal transition, with increased urbanization, mixed marriages, and the use of technology in communication creates room for greater independence of women and less violence. However, IPV may increasingly be used as a control mechanism when facing new expectations of what it means to be a man or a woman [17,25]. Inequalities between men and women persist and are often covered by referring to gender norms and traditions, including violence [15]. Jewkes [18] has argued that in many countries use of physical violence is normatively accepted but that there is a clear limit to the tolerated severity of violence. Studies from Nigeria and Uganda indicate that these norms become internalized and the women themselves believe that a man has the right to beat his wife under certain circumstances such as when she does not complete household work adequately, refuses sex, disobeys her husband, or is unfaithful [29,30]. A socio-ecological analysis of data from 17 countries showed that women were two times more likely to justify physical violence than men [31]. For Tanzania, the WHO multi-country study reports similar results with more than 60% of women in urban areas believing that a man can have a reason to beat his wife [32].

### Theories on causes and consequences of IPV against women

There are several theories about the causes and consequences of IPV against women that are relevant for this study. *Culture of violence *theory focuses on gender related norms that permit use of violence by a dominant group to others. IPV is seen more frequently in societies where men are considered superior and dominant [33]. The theory emphasizes the increased risk of violence in societies where violence has become integrated into the culture [18,26,28]. *Power theory *takes this a step further by pointing specifically to the influence of power in violent relationships. Levinson's [33] ethnographic study identified four elements of power. Economic inequality between men and women; use of IPV to control the family; men's authority; decision making powers and bureaucracy in the divorce process as determinants of domestic violence. *Feminist theory *[34] relates IPV to the power of men as the dominant class. Men have more access to symbolic and material resources than women who are devalued, secondary and inferior to men. IPV against women is therefore less important because male dominance influences all aspects of life. If the power relation is threatened, IPV may increase due to conflicting expectations of masculinity [18]. However, in societies where women's status is very high or very low, the level of IPV may be low since violence has no role in reinforcing male authority. Where sanctions are strong and functional (legal or cultural), IPV can decrease because of controlled violence within those societies. *Social learning theory *focuses on the social context where behaviour is a result of observational learning, modelling and imitation [35,36]. Men's violent behaviours are a result of their own previous experience of violence or of having witnessed their mothers being abused [37,38]*. The ecological model *[39] offers a comprehensive understanding of risk factors linked to IPV at different levels. The individual level indicates biological characteristics and other individual experiences related to gender norms and expectations which predict men who will perpetuate IPV towards their women partners in many settings [37,38]. Other individual IPV risk factors build on social learning theory and include witnessing violence or being abused as a child or as an adolescent [19], having partners who are excessive alcohol or drug users [7,37,39], or being financially dependent [40,41]. The relationship level refers to the immediate context in which abuse may occur, for example, male control over family resources, decision-making power, economic inequalities, and high levels of controlling behaviours [21,32,42-44]. The community level is extended to family, neighbours, work and other social networks. Risk factors include restrictive marriage norms [19], honour killings [45] and lack of social support from others due to the silence associated with violence [18,27]. The societal level includes dominant societal norms, laws and socio-economic policies that may influence sanction mechanisms. Lack of specific policies and laws to protect IPV-affected women and lack of adequate sanction mechanisms for perpetrators are risk factors for IPV [18]. Accepting and practicing polygamous relationships has also been linked with IPV against women in sub-Saharan Africa [20,46].

In a previous study, we focused on the experiences of healthcare workers who meet women clients exposed to IPV against women in Temeke District, Dar es Salaam, Tanzania. The health care workers perceived IPV against women to be linked with gender inequality, male dominancy, and poverty. They could identify substantial barriers for clients to disclose their violence experiences, but were concerned and eager to make a difference if provided with adequate skills, tools and resources [27]. However, to be able to suggest relevant and feasible community- and health care based interventions there is a need to explore further community member understanding and response towards intimate partner violence.

The specific objectives of this study were to explore how community men and women understand IPV and to capture their views regarding social, medical and legal support of IPV survivors as well as their suggestions for preventive measures.

## Methods

### Design

**Figure 1:**
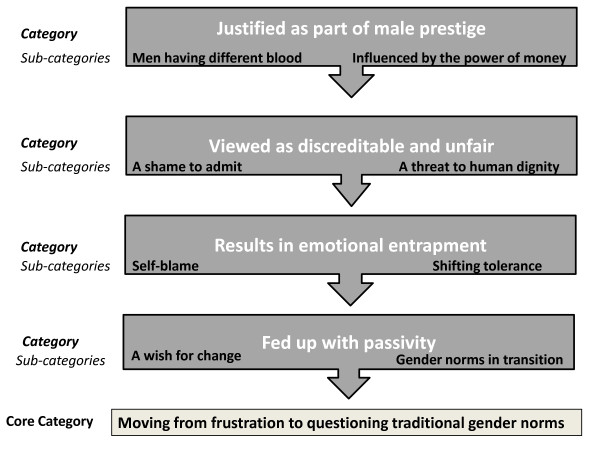
**A model showing the relationship of the core category, categories and their corresponding sub-categories**.

We conducted a qualitative study based on focus group discussions (FGDs) with community members. FGDs are suitable for generating information on how norms, systems and attitudes are formed and how they differ between groups in a community. The data were analysed using the steps for grounded theory [47]. The findings were summarised in a model (Figure 1) and later integrated into the existing theoretical framework for understanding IPV against women.

### Setting

The study was performed in Temeke District, one of three districts in the Dar es Salaam region of Tanzania. The Temeke District population is approximately 770,000 [22] with an annual growth rate of 4%. Eighteen per cent of the population lives below the poverty line. Temeke is a semi-urban district with a mix of Tanzanian tribes including both Muslims and Christians. The district consists of 18 wards of which four are peri-urban. Each ward is divided into streets that are headed by a local government leader[48]. The basic traditional gender norm assumption in this setting, as in other parts of Tanzania, is that a woman's primary commitment is to care for her family at home and to rely on a male breadwinner who is responsible for cash and other household needs. The skills of these self-employed housewives tend to be undervalued and defined as unskilled, even when they entail complex actions and thought processes such as child care, subsistence farming, agro-processing. Customary laws and practices remain discriminatory against women on issues of property inheritance particularly for land, and in decisions on the consequences of actions such as IPV [48]. Christians have strong beliefs that marriage is a life commitment and this increases acceptance of IPV and makes it hard for women to leave violent relationships. Strict Muslim divorce procedures further increase the likelihood of Muslim women tolerating violent relationships. According to Tanzanian law, physical violence is a criminal offence. However, IPV against women is not considered separately and the law on sexual assaults does not currently include rape within marriage [24].

### Selection of informants

We purposively selected our informants from the Temeke District hospital catchment area. The Temeke District management helped select four peri-urban wards of the 18 district wards. To minimize travel, we randomly selected one of the four wards since there was no socio-economic variation expected among them. Three streets within this ward were randomly picked from a list of streets given by the local government leaders. The local leaders were more familiar with the surroundings, so they assisted us in approaching community members who could capture the demographic and socio-cultural variation in the area. The specification was that community members should be between 15-59 years of age and likely to have met with people affected by IPV at work or in their community. Local leaders provided us with a list of names, indicating professions for those employed and business type for the self-employed or unemployed. A member of the research team (RL) purposively picked names to ensure variation in sex, age and profession (teachers, accountants, secretaries and agricultural officers), including religious leaders. The final sample of informants is shown in Table 1.

**Table 1:** Characteristics of the focus groups participants (n = 75)

Group number	Sex	Category	Number	Age (years)
1	Females	Non-professionals	12	15-40
2	Males	Non-professionals	12	30-48
3	Females	Professional and non-professionals	12	30-52
4	Females	Professionals	10	30-46
5	Males	Professionals	11	27-56
6	Females and males	Religious	10	35-52
7	Males	Professionals and non-professionals	8	32-41

### Data collection

Data were collected for four months and the FGDs were moderated by two researchers (a man and a woman) who were working with studies on violence. After introducing ourselves to the focus group participants, we opened the discussion by showing a drawing of a woman who looked sad. The discussants were asked to reflect on possible reasons for her state without first mentioning IPV. Later, we showed them more specific newspaper headlines, such as "What does it mean when a father beats his wife in front of the children?" to initiate a discussion on IPV and its consequences. We used a thematic guide (Additional file 1) to help us probe further.

Informed consent was obtained before each focus group and we asked for permission to record and take notes to be able to capture the interaction between participants. Each focus group had 8-12 participants. They were conducted in a quiet venue within the informants' neighbourhood and each lasted 65-90 minutes. After every FGD, we reviewed and discussed the collected information so that any new insights could guide subsequent discussions, a technique called emergent design [46]. After the seventh FGD, we felt that we had reached saturation, a state where additional information was not expected to give new insights. One of the groups was comprised of Christian leaders only since Muslim leaders were well represented in the other groups.

### Research tools

We prepared a thematic guide (Additional file 1) with four general themes. These were social cultural factors; policy environment; risks, and help-seeking behaviours in violent relationships; and future expectations of care, support and IPV prevention. The specific areas included community awareness and experiences of IPV as well as gender norms and attitudes towards marriage, IPV consequences, family, social, medical and legal support, elder and local governmental leader roles and participant suggestions for prevention. Development of the guide was based on a general understanding of IPV. During data collection and initial analyses this pre-understanding was put within brackets [47].

### Analytical procedures

FGDs were transcribed verbatim in Kiswahili, which is the official language in Tanzania. Later they were translated into English to enable joint analysis by the research team. Following a grounded theory approach the text was imported into the Open Code 2007 program [49] to facilitate the coding process. After reading the transcripts, RL and ME performed open coding of the text, constantly comparing similarities and differences by going back to the original text. In the next step, RL and ME performed selective coding where relevant codes were further conceptualized and leading to the development of four main categories and eight sub-categories relevant to our research focus (Figure 1). On the basis of these categories and sub-categories, a core category was constructed to capture the essence of the findings. In a last stage the analysis was integrated and compared with existing theory.

### Ethical considerations

We obtained ethical clearance from Muhimbili University of Health and Allied Sciences (MUHAS) in June 2007. Next we received permission from the local council to conduct the study in the selected wards. In accordance with the ethical guidelines of research on violence against women [50], we introduced the study as a "study on women's health and life experiences". To protect the participants from possible suffering by discussing sensitive issues, we established linkages with service providers such as Dar es Salaam Crisis Centre and Tanzania Legal Women Association (TAWLA), and appointed a psychiatric nurse to give support if needed. During the introductory part of each FGD the participants were encouraged to agree to keep the information discussed within the group. Discussants who reacted emotionally when recalling experiences of IPV were politely invited to leave the discussion and supported by one of the team members until they recovered and decided to return. None of the participants wanted to be referred to a counsellor.

### Trustworthiness

Two of the research team members, RL and HL, are Tanzanians and fluently speak the local language of Kiswahili. The other research team members (LN and ME) reside abroad and have extensive experience in cross-cultural collaboration. To increase credibility, the research team made repeated visits to the study site. Prolonged engagement in the field by RL helped to build trust with the community representatives. Preliminary findings were subjected to member-checks with two Temeke district residents to confirm meanings of certain local expressions. The research team also had continuous peer-debriefing sessions as the study progressed. A flexible guide, emergent design, multidisciplinary research team, verbatim transcriptions, and predefined analytical procedures were used to promote study rigour. During the analysis, the fitness and relevance of emerging categories to the research question were tested by constant comparison and checking between the text, codes and categories and by paying specific attention to outliers or negative cases.

## Results

Analysis of the focus group discussions resulted to one core category, **"Moving from frustration to questioning traditional gender norms"**, denoting a community in transition where the effects of intimate partner violence in the community had started to fuel wishes for change. The community members had started questioning the role of existing gender norms and IPV against women. The core category emanates from eight sub-categories: two for each of the four categories as illustrated in Figure 1.

### 'Justified as part of male prestige'

The first category, '**Justified as part of male prestige**', denotes a socially constructed gender norm system where men are born to be more powerful than women, are given a superior position in decision making, and believed to have the right to a better financial status and position compared to women.

#### Men having different blood

The sub-category *Men having different blood *indicates ideas of masculinity seen to influence partner/husband behaviours and perceptions of IPV against women. Both women and men of all ages and professions justified a certain level of IPV against women. Men were allowed to use violence as a measure to correct women in specific situations and women accepted a certain amount of violence for discipline. Men expressed their preference for women who acted quietly, asked for permission to go out, were obedient, and had few friends. To their understanding, younger women who violated social norms were more exposed to violence. Both men and women described how men's dominance and pride made them annoyed and threatened by non-obedient women who shouted or gave men instructions. They mentioned inability to care for children, being late from work, food not ready in time, or refusal for sex as examples of situations that justified violent acts. They also indicated a clear limit for the level of acceptable violence. In the men's group, emphasis was put on presumed sex differences.

D6: *"We men are proud. We do not want to be given instructions by women but want to instruct. That is the way we were brought up. We have different blood; unlike women who use words, we cannot wait to beat*." {FGD 5}

D1: "*Men cannot just have a mere talk when they are annoyed, as head of the family, a slap or two is okay to your wife, but not bloodshed." *{FGD 5}

Discussants from a women's group justified beating in similar ways and there were often stories about how other women intentionally provoked men.

D4: *"We annoy our husbands with our behaviours and sometimes we deserve to be beaten. I hear some women from certain tribes provoke men to beat them feeling that beating is part of love...." *{FGD 3}

#### Influenced by the power of money

The sub-category *Influenced by the power of money *emphasized the burden that financial power put on both men and women and expressed how dependency on men for their daily living influenced women to stay in violent relationships.

D6: "*A man who does not have good money is not loved, while a rich husband may beat his wife at night and in the morning pledge her with offerings and presents. Many women continue to stay with abuse in this way." *{FGD 4}

Few male discussants noted the existence of IPV perpetrated by women. Those who had low or no income were said to be at risk of both verbal and physical abuse and sometimes divorce, since they did not comply with the norm of being breadwinners or the community expectation of men being "heads of their families".

D5: "*I used to live better when I had a job. The problems started when I left the job. I suffered from abusive words and sometimes slaps from the woman I used to love. I tried to give her hope for our future but she was not ready to listen because I had no money." *{FGD 2}

D2: *"As for me, despite her abuse I tried to convince her to come back after she left me with the children, but she refused because I had no money. I suffer joblessness and abuse at the same time. I hear she lives with a shopkeeper in the next street." *{FGD 2}

### 'Viewed as discreditable and unfair'

The second category, '**Viewed as discreditable and unfair**', stood out when male and female informants talked about the effects of daily and long-term IPV. They mentioned the unfair situation that resulted primarily from men being violent, irresponsible, and not supporting their family's needs. There were many stories about married men arriving home late after having spent most of their earnings on other women.

D3: "*I have witnessed several men who spend their money on other women and leave their families to suffer. Most times they don't even eat at home. If a woman questions anything, she is given "presents" of slaps and verbal abuse. These women are always miserable and angry." *{FGD 3}

In men's FGDs such behaviours were noted to be unfair too for the women and they emphasized the misery, misfortune, and unhappiness resulting from long exposure to IPV.

D2: "*One of my neighbours abuses his wife after being late so that the wife does not get a chance to question him; instead she concentrates on the pain and fear. This woman is always miserable." *{FGD 6}

#### A shame to admit

*A shame to admit *emerged when women expressed their difficulties of disclosing IPV experiences that led to isolation, helplessness and limited social support from friends and the community. They also described the stigma and disgrace that abused women are believed to cause their families, friends and neighbours. Affected women were said to hide and avoid IPV disclosure to reduce pain for themselves and others.

D6: *"My friends used to laugh at me; some suspected that I was living in an unhappy marriage. Actually I did not enjoy sex and hated men. I felt embarrassed and it was shameful to pass in front of others on the streets. I looked ugly and had rough skin in those days*." {FGD 1}

D7: *"I cried several times, alone in my bed room...until we divorced. It was not easy to inform my father-in-law or anybody; the situation was too shameful." *{FGD 1}

#### A threat to human dignity

*A threat to human dignity *came up when both female and male informants mentioned the universal needs for empathy, solidarity and support. People affected by IPV were seen as being denied these basic human rights. In their discussions, women wanted men to understand that women also "have flesh" and "feel pain". Those who shared own experiences talked about being rejected, disliked and not respected by their families or society.

D6: *"But even if you are beaten once in your life you feel pain, and it is not easy to forget. My friends used to laugh at me. Other colleagues and distant family members were abusing me by saying that I was beaten because I was poor at making love. I had frequent disagreements with my husband with several injuries, sad moments and sleepless nights." *{FGD 1}

Men also mentioned experiences of verbal abuse, being denied sex or accused of not being good bread earners as threats to their dignity.

D5:*"I invested in a shop and gave the responsibilities to my wife. When the business grew she got a boyfriend from the place she collects wholesale products and started to abuse me. Because of my children, I am living with my wife but with precautions. I sleep in a separate room. Suffer silently." *{FGD 2}

### 'Results in emotional entrapment'

The third category, '**Results in emotional entrapment**', emanates from a Kiswahili metaphor that refers to marriage as a trap ("ndoa ndoano"). This was mentioned as an explanation of how violent relationships make individuals feel trapped in their marriages and unable to decide to leave, despite abuse.

#### Self blame

A sub-category, *Self-blame*, shows how abused women blame themselves for the violence and became disoriented. The informants described how affected individuals turned fearful and withdrew from social life:

*D5: "It is difficult to advise somebody who is living with violence to move out. She would always complain and blame herself because she loves the man. I wonder why a person who has problems with violence stays. Maybe she stays for the sake of the children but also for the shame, or for fear of living alone." *{FGD 4}

#### Shifting tolerance

*Shifting tolerance *describes a process where, at a certain point, women start to look for help or revenge in an effort to escape or at least receive help in their relationship. This process was said to take time since continuous stress and physical aggression alter the woman's perceptions of reality and what marriage means.

*D2:"He threw nasty words and I packed and stayed with my friends for three days. He asked my father for help to get me back but I said that I suffered from seeing my mother being slapped and that was enough. But on the third day I felt that I had to go back to the perpetrator, I mean my husband." *{FGD 4}

Many of the coping mechanisms mentioned were emotionally focused, including decisions to fight back or to shame the abuser by revealing the violent behaviour beyond family members to co-workers or friends. Other ways to reduce the harm or end the violence were fighting back, meditation or engaging in religious activities.

### 'Fed up with passivity'

The last category, '**Fed up with passivity**', expresses that both men and women are tired and annoyed with IPV.

#### A wish for change

Both men and women expressed *A wish for change *and underscored women's role in this change.

D2: "*We need to change, show that women don't accept violence any longer." *{FGD 4}

D6: *"The time to be men's instruments should stop. They need us just as we need them. They continue their bad habits because we are quiet. This is not good enough." *{FGD 1}

Experiences of abusive relationships were discussed openly in the FGDs and indicate that IPV is common. However, at the individual level the informants emphasized that actions to prevent IPV are possible and that the silence surrounding it has to end.

D6: "*After three years of suffering I decided to end the silence and shared the dangers I was living in with my brother who assisted in divorce. It was not possible to make it without his support." *{FGD 4}

#### *Gender norms in transitio*n

The sub-category *Gender norms in transition *emerged when men started to reflect on their current role in maintaining gender norms that justify violence.

D1*:"IPV should stop; you know the women we beat are other people's sisters and mothers." *{FGD 5}

This statement triggered further discussion on differences and similarities between violence against intimate partners and other family members. Both men and women agreed there was a need for action at many levels and that the current harmful gender norms must be changed. They mentioned the need for consolidated efforts at different levels, governmental as well as health care.

D4: *"I think the solution to intimate partner violence problems should come from above. I mean they should be initiated by the cabinet as a higher level government organ. We may fail to solve matters individually. We witness cases of violence and they are reported in the newspaper but we are not aware of what goes on thereafter. I think we should also involve health care...." *{FGD 5}

Some informants reflected on the importance of supportive neighbours but clarified difficulties for community members to intervene in complex situations that involved violence. They saw the need for more structural changes including strong laws that could clearly indicate a normative change in attitude towards IPV against women.

D6*: "People fight at night with the doors closed. Who will open the door for you? Can you break the door of somebody's house? It is an offense. Maybe what we need are strong laws which can strictly be followed." *{FGD 7}.

## Discussion

**Figure 2:**
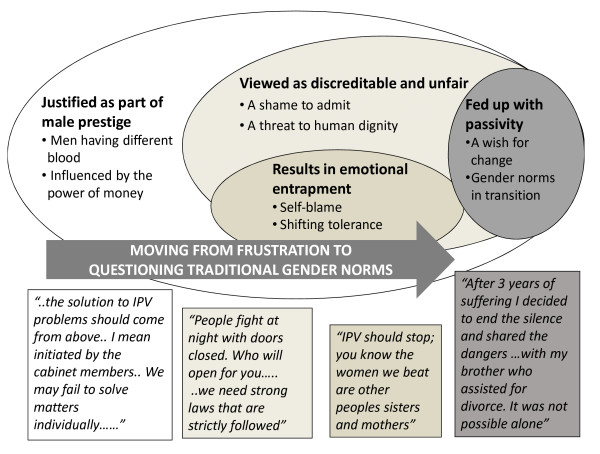
**Linking the results to the ecological framework**. {Societal (white ellipse), community (light grey ellipse), relationship (dark grey ellipse), and individual (darkest grey ellipse) levels of the ecological framework. Core category = white text within the arrow, categories = bolded, sub-categories = bulleted. Quotations (in boxes).

Our findings denote a community in transition, where the effects of intimate partner violence have started to fuel a wish for change. Consistent with the ecological model developed by Heise [39], the results indicate linkages between individual, relationship, community and societal influences for both understanding and response to IPV against women. Hence, this discussion will follow the ecological model which gives a comprehensive and important framework for illustrating community perceptions of violence against women, including IPV [39]. See Figure 2.

### Societal level

According to the ecological model [39], factors acting at the societal level include norms of masculinity linked to male dominance, laws granting men control over women's behaviours, and attitudes that accept men's violence as a way to resolve family conflicts, and provision of inadequate policies and structures to address IPV [5,27,51,52].

Our findings that IPV is '**Justified as part of male prestige**' fits well at the societal level of the ecological framework and indicates how the concept of masculinity is built on social norms that value men as superior and more powerful than women. These norms subordinate women in many life spheres, from economic independence to lack of decision-making power [21,40,53]. This happens when societal norms allow the use of IPV to reprimand women and where men are expected to have the final say in the use of family resources [5,28].

Previous studies show how patriarchal norms influences violence. Wife beating is more common in households where power is concentrated in the hands of the husband or male partner [23,54]. In these households, physical violence may be used to legitimize the dominant position of men while at the societal level, cultural norms allow men to use violence in order to maintain control.

In our study, views about masculinity and femininity considered men to *have different blood*. This became part of community member explanation and justification for violence to a certain degree and as a way to normalize the situation. Gender-constructed norms were illustrated in reports that some women would even provoke IPV from their husbands/partners and this was seen as an indication of love. The WHO multi-country study also showed that a large proportion of Tanzanian women justified both physical and sexual violence under certain circumstances [55]. Negussie et al [21] showed similar results from Ethiopia, however rural, illiterate women were more likely to believe that men had a right to beat and were less likely to think that they could refuse sex than urban, literate women. In a study from Dar es Salaam, we also found that healthcare workers internalize cultural beliefs and practices that support subordination of women and allow men to use IPV as a disciplinary tool [27].

In the present study, the sub-category *Influenced by the power of money *indicates a male dependency where women are unable to manage life alone. The low socio-economic status of women and lack of support from peers are seen as important factors that are part of the explanation of continued IPV against women as well as possible entry points for interventions [46]**. **A Nicaraguan study by Valladares [15] also linked IPV to deep rooted gender inequalities such as lack of decision-making power and access to own resources among women. However, others have found that high economic status of women compared to their partners increases IPV [56]. This discrepancy can be explained by normative roles among men and women in patriarchal societies where men dominate. When women are empowered to support the family economy, their normative roles of being housewives expected to do domestic work is defeated.

### Community level

At the community level, low social capital, lack of institutional support (religious, police, medical), poverty and related factors, and social environment supportive of IPV explain increased or decreased IPV.

In our study, community members were unhappy with the existence of IPV as indicated by the category '**Viewed as discreditable and unfair**'. We relate this to the concept of social stigma that is known to strongly affect health. Jacoby et al [57] state that "when the societies categorize individuals into certain groups, the stigmatized are subjected to status loss and discrimination". The dimensions of social stigma related to IPV in our study extend to people outside the family sphere and affect social interactions. These are potential additional threats to health and a barrier to preventive efforts.

Women exposed to violence fear being stigmatized by colleagues and family members. They have difficulty in being open about their IPV experience as shown by our sub-category *A shame to admit *which also meant a failure to seek community, legal and medical support. These findings are supported by a cross-sectional study in Nordic countries [58] where less than 10% of women who experience physical violence from their partners revealed this to their gynaecologist. Many health institutions are challenged to provide medical support to women who do not disclose IPV and this makes it hard for them to give adequate care. In a previous study on experiences of IPV, healthcare workers struggled to elicit IPV information from their clients [27].

Our findings also indicate a *Human rights *concern which refers to the "basic rights and freedom to which all humans are entitled" [59]. Despite global acknowledgement that IPV is a basic human rights violation, this is not explicit in Tanzanian law [24,60]. In the study area, a woman's level of understanding about legal issues is unclear. Education and awareness of the existing legal system has not reached the majority of women. In our previous study, we found that women were discouraged from reporting IPV to the police. Unfavourable structures and legal procedures were mentioned. Informants recalled many stories from women who experienced bribe requirements and unfair treatment from the police. This led women to lose trust in the legal system when it came to IPV [27]. In the present study, informants viewed IPV as *A threat to human dignity*. This concern is important and a basic step to decrease of IPV against women in the studied community.

### Relationship level

The relationship level of the ecological model includes issues of marital conflict mostly due to male control over wealth and decision-making. In the current study, discussants were concerned about the consequences of violence for women as summarized in the category '**Results in emotional entrapment**' accompanied by *Self-blame *and *Shifting tolerance*. Our study illustrates how survivors use coping mechanisms such as engaging in religious activities or attempts to fight back for temporary safety. These results can be linked to the high prevalence of controlling behaviours described in the WHO multi country study where 90% of ever-partnered women in an urban Tanzanian setting experienced one or more controlling behaviour. The likelihood of having experienced violence increased with the number of controlling behaviours by the partner [18]. Negussie et al [21] show that the risk of depression is related to level of controlling behaviour.

### Individual level

The individual level of the ecological framework refers to individual risk factors such as being male or a female, alcohol and substance use, employment status, educational level and previous experience of abuse. In our study, men's alcohol abuse and lack of employment were mentioned as closely linked with violent behaviour. These findings are similar to the report of Kolawole & Uche [28] in their study of women's perceptions about domestic violence in Nigeria. Our discussants also mentioned the link between men having multiple partners and physical violence. These results are supported by Anderson et al [61] in studies from eight southern African countries, and by the study of Lawoko et al of social inequalities in IPV among Kenyan women [23].

Men's violent behaviours are associated with having witnessed abuse and IPV earlier in life [35]. The theory of learned behaviour emphasizes that violence should be seen as a learned behaviour based on the construction of gender norms. At the individual level, aggression can emerge from frustration over a man's inability to control the female partner [62], an area not frequently discussed in the FGDs. However, our findings show that men who are unable to fulfil their expected gender roles as bread winners suffered IPV from their wives/partners. The women were said to engage in relationships with other men or use violent acts towards their husbands/partners. Such gender norm sequelae are important issues to recognize and address and point to the need for engaging men in strategies to prevent gender-based violence.

Our model, shown in Figure 2, does not specifically address the individual level risk factors for violent behaviours as postulated by Heise [38] but focuses on the strong reactions against violence that were expressed in all of the focus groups. Both men and women were tired of seeing women and children suffer and were **'Fed up with passivity**'. Many community members had started to question the norms that allow violence to be accepted, and at the same time feel trapped by the power of internalised traditional gender norms. This illustrates how transition in norms at a societal level causes tension at the individual and community levels [18,39]. Some he suggested actions included that the government have a greater commitment by involving cabinet members, healthcare, judiciary systems and local governments. These recommendations are similar to those from a study in Vietnam where community members pointed out the need for the health and other governmental sectors to initiate preventive efforts [15]. Also, this is in accordance with WHO recommendations to involve the health sector, create programs for improved gender norms, provide micro credit interventions, and strengthen the use of laws and policies in order to decrease IPV prevalence [28]. A WHO report [63] points out that the elimination of violence against women is not specifically targeted in the Millennium development goals. Goal 3, "promote gender equality and empower women" needs to be expanded with a clear target of reducing lifetime IPV prevalence since violence against women hinders progress in achieving goal 3 and all other Millennium goals. The report also underscores that the elimination of violence requires targeting the gender-based norms that exacerbate discrimination against women. Since the determinants of IPV are complex and vary between cultural settings and levels, there is a need for countries like Tanzania to be more pro-active in research that evaluates the relevancy and applicability of different preventive approaches [64]. Studies to evaluate strategies that emphasize primary prevention, target behavioural and attitudinal change at younger ages, and engage both men and women in challenging harmful gender norms are specifically called for [18,65].

### Strengths and limitations of the study

Among the strengths of the study are the methodological efforts to achieve a diverse and representative sample of informants from the community and the rigour of the coding and analysis phases. The use of focus groups for data collection may have given an overly positive view of the transition in gender norms if participants avoided expression of deviant views that justify violence. We think that the discussions were open, free and reflected an increasing awareness about the seriousness of gender-based violence. We also noted the complexity of the situation with community members who are trapped within traditional expectations of masculinity and femininity and at the same time realise the harmful consequences of those expectations. While the study results cannot be generalized to all areas of Tanzania (or Africa) they are in line with many other studies from settings characterized by similar gender norm systems [18,19,21,23,53,61].

## Conclusions

Prevailing gender norms still accept that women are in a subordinate position with limited possibility to control their own lives. Thus, they remain at continued risk for IPV against women. Community members have started to question these norms because of their negative effects on women's health. Hence, the Tanzanian government and other governments with similar situations need to show an increased commitment and prioritize IPV preventive efforts. This can be done by having healthier policies and resources to re-enforce legal rights as well as providing adequate medical care for IPV survivors and perpetrators. At the community level, it is necessary to raise consciousness about the human rights perspective of women's position, challenge gender norms that perpetuate IPV, and advocate for an IPV-free society. The authors acknowledge an increased interest in violence issues from the media and from government and non-governmental associations in Tanzania. However, our study reveals that these efforts need to be multiplied and focus societal-, community-, relationship, - and individual level approaches by deliberately engaging men.

## Competing interests

The authors declare that they have no competing interests.

## Authors' contributions

RL was involved in the design of the study, data collection, transcription, analysis and drafting of the manuscript. ME was involved in the design of the study, analysis and drafting of the manuscript. LN was involved in the design of the study and in drafting of the manuscript. HL participated in the translations of FGDs and drafting of the manuscript. RL, ME, LN and HL had access to the data and are responsible for its integrity. All authors read and approved the final manuscript.

## Pre-publication history

The pre-publication history for this paper can be accessed here:

http://www.biomedcentral.com/1472-6874/11/13/prepub

## Supplementary Material

Additional file 1 **Key points/issues which were considered to guide the discussions when obtaining information on community understanding and response to IPV**. Click here for file
