# Supplementary material for: Community perceptions of intimate partner violence - a qualitative study from urban Tanzania
Source: BMC Womens Health. 2011 Apr 18;11:13. doi: 10.1186/1472-6874-11-13 (PMC3094305; doi:10.1186/1472-6874-11-13)
Supplement: Additional file 1 — Key points/issues which were considered to guide the discussions when obtaining information on community understanding and response to IPV. [file 1472-6874-11-13-S1.DOC]

## Additional file 1

- **Social cultural factors related to IPV**
  - Awareness of IPV in the community
  - Values in married that influence IPV
  - Myths and cultures to wife battering
  - Provocative factors
  - IPV effects/family, children, affected people, men

####

- **Policy environment**
- Policy awareness and suggestions
- Elders and local government leaders roles
- **Risks and health/help seeking behaviors in violent relationships**
  - Where and to whom women go for care/report about IPV
  - How women/others respond to situation, home, judiciary, healthcare, local leaders
- Perceived risks factors by self/others, future
- Suggestions for prevention and support
- Personalities among men and women
- **Future expectations of care, support and IPV prevention.**

***Ending the discussion***

Is there anything that you think we have not discussed and that you would like to add? If so please feel free to do so....

Thank you very much for your participation. Your input has been very valuable. The study results will later be disseminated to different stakeholders, including at the community level.

Thanks… Bye
